# {2-[(2-Hy­droxy­benzyl­idene)amino]-4,5,6,7-tetra­hydro-1-benzo­thio­phen-3-yl}(phen­yl)methanone

**DOI:** 10.1107/S1600536814006199

**Published:** 2014-03-26

**Authors:** Manpreet Kaur, Jerry P. Jasinski, Channappa N. Kavitha, H.S. Yathirajan, K. Byrappa

**Affiliations:** a Department of Studies in Chemistry, University of Mysore, Manasagangotri, Mysore 570 006, India; b Department of Chemistry, Keene State College, 229 Main Street, Keene, NH 03435-2001, USA; c Materials Science Center, University of Mysore, Vijyana Bhavan Building, Manasagangothri, Mysore 570 006, India

## Abstract

In the title compound, C_22_H_19_NO_2_S, the cyclo­hexene ring adopts a slightly distorted half-chair conformation. The dihedral angles between the mean planes of the thio­phene ring and the phenyl and 2-hy­droxy­phenyl rings are 70.4 (5) and 12.1 (9)°, respectively. The phenyl and 2-hy­droxy­phenyl rings are twisted with respect to one another by 81.0 (6)°. A short intra­molecular O—H⋯N hydrogen bond is observed. In the crystal, weak C—H⋯O inter­actions link the mol­ecules into zigzag chains diagonally along [100] .

## Related literature   

For the importance of thio­phene derivatives, see: Molvi *et al.* (2007 ▶); Rai *et al.* (2008 ▶); Asthalatha *et al.* (2007 ▶). For applications of 2-amino­thio­phene derivatives, see: Sabnis *et al.* (1999 ▶); Puterová *et al.* (2010 ▶); Cannito *et al.* (1990 ▶); Nikolakopoulos *et al.* (2006 ▶); Lütjens *et al.* (2005 ▶). For the biological and industrial importance of Schiff bases, see: Desai *et al.* (2001 ▶); Karia & Parsania (1999 ▶); Samadhiya & Halve (2001 ▶); Singh & Dash (1988 ▶); Aydogan *et al.* (2001 ▶); Taggi *et al.* (2002 ▶). For a related structure, see: Kubicki *et al.* (2012 ▶). For puckering parameters, see Cremer & Pople (1975 ▶). For standard bond lengths, see: Allen *et al.* (1987 ▶). 
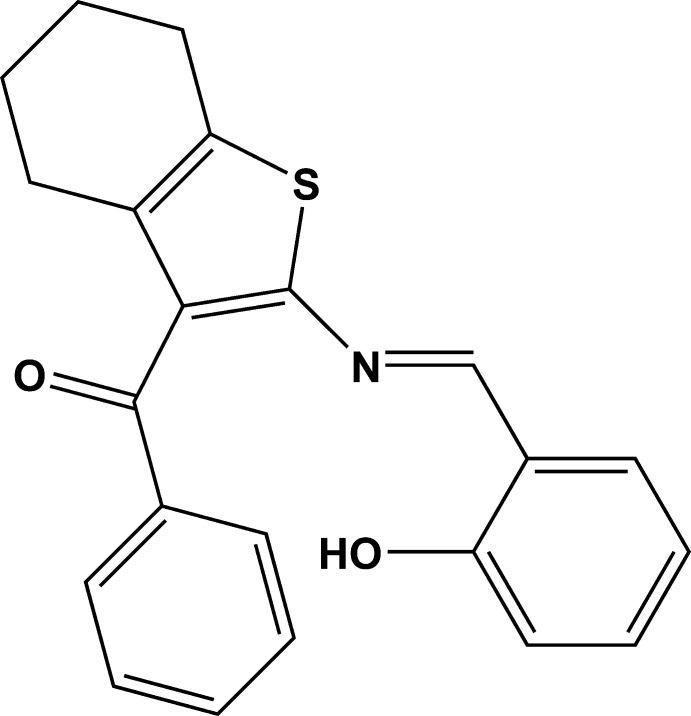


## Experimental   

### 

#### Crystal data  

C_22_H_19_NO_2_S
*M* *_r_* = 361.44
Monoclinic, 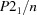

*a* = 9.26395 (15) Å
*b* = 14.2886 (2) Å
*c* = 13.6476 (2) Å
β = 96.7581 (15)°
*V* = 1793.97 (5) Å^3^
*Z* = 4
Cu *K*α radiation
μ = 1.73 mm^−1^
*T* = 173 K
0.24 × 0.14 × 0.08 mm

#### Data collection  

Agilent Eos Gemini diffractometer
Absorption correction: multi-scan (*CrysAlis PRO* and *CrysAlis RED*; Agilent, 2012 ▶) *T*_min_ = 0.658, *T*_max_ = 1.000
11611 measured reflections
3462 independent reflections
3113 reflections with *I* > 2σ(*I*)
*R*_int_ = 0.053

#### Refinement  

*R*[*F* ^2^ > 2σ(*F* ^2^)] = 0.049
*wR*(*F* ^2^) = 0.142
*S* = 1.06
3462 reflections
237 parameters
H-atom parameters constrained
Δρ_max_ = 1.03 e Å^−3^
Δρ_min_ = −0.26 e Å^−3^

### 

Data collection: *CrysAlis PRO* (Agilent, 2012 ▶); cell refinement: *CrysAlis PRO*; data reduction: *CrysAlis RED* (Agilent, 2012 ▶); program(s) used to solve structure: *SUPERFLIP* (Palatinus & Chapuis, 2007 ▶); program(s) used to refine structure: *SHELXL2012* (Sheldrick, 2008 ▶); molecular graphics: *OLEX2* (Dolomanov *et al.*, 2009 ▶); software used to prepare material for publication: *OLEX2*.

## Supplementary Material

Crystal structure: contains datablock(s) I. DOI: 10.1107/S1600536814006199/sj5393sup1.cif

Structure factors: contains datablock(s) I. DOI: 10.1107/S1600536814006199/sj5393Isup2.hkl

Supporting information file. DOI: 10.1107/S1600536814006199/sj5393Isup3.cml

CCDC reference: 992723

Additional supporting information:  crystallographic information; 3D view; checkCIF report

## Figures and Tables

**Table 1:** Hydrogen-bond geometry (Å, °)

*D*—H⋯*A*	*D*—H	H⋯*A*	*D*⋯*A*	*D*—H⋯*A*
O2—H2⋯N1	0.82	1.92	2.641 (2)	146
C18—H18⋯O1^i^	0.93	2.51	3.436 (2)	174

Symmetry code: (i) 
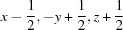
.

**Fig. 1.**
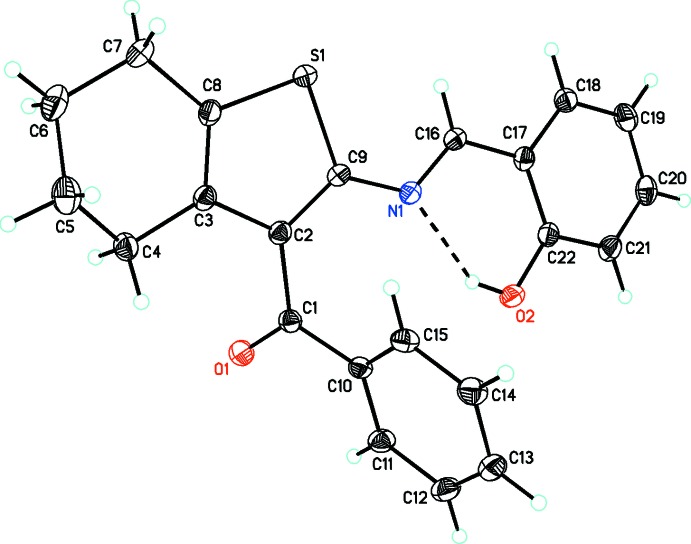
ORTEP drawing of (I) (C22H19NO2S) showing the labeling scheme of molecule with 30% probability displacement ellipsoids. The dashed line indicates a short O2—H2···N1 intramolecular hydrogen bond.

**Fig. 2.**
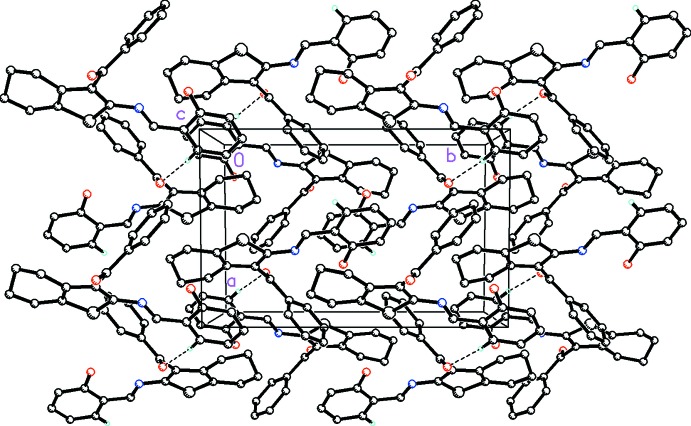
Molecular packing for (I) viewed along the c axis. Dashed lines indicate weak intermolecular C—H···O interactions which link the molecules into zig-zag chains along [101]. H atoms not involved in these weak intermolecular interactions have been removed for clarity.

## supplementary crystallographic information

### 1. Comment

Thiophene derivatives have been reported to exhibit a broad spectrum of biological properties such as anti-inflammatory, analgesic, antidepressant, antimicrobial and anticonvulsant activities (Molvi *et al.*, 2007; Rai *et al.*, 2008; Asthalatha *et al.* , 2007). 2-Aminothiophene derivatives have been used in a number of applications as pesticides, dyes and pharmaceuticals. Reviews of the synthesis and properties of these compounds have been reported (Sabnis *et al.* 1999; Puterová *et al.* 2010). Substituted 2-aminothiophenes are active as allosteric enhancers at the human A1 adenosine receptor (Cannito *et al.*,1990; Nikolakopoulos *et al.*, 2006; Lütjens *et al.*, 2005). Schiff base compounds are an important class of compounds both synthetically and biologically. These compounds also show biological activities including antibacterial, antifungal, anticancer and herbicidal activities (Desai *et al.*, 2001; Karia & Parsania, 1999; Samadhiya & Halve, 2001; Singh & Dash, 1988). Furthermore, Schiff bases are utilized as starting materials in the synthesis of compounds of industrial (Aydogan *et al.*, 2001) and biological interest such as β-lactams (Taggi *et al.*, 2002). The crystal and molecular structures of two 2-aminothiphenes have been previously reported by our group (Kubicki *et al.*, 2012). In continuation of our work on 2-aminothiophenes and Schiff bases, we report here the crystal structure of the title compound, (I), C_22_H_19_NO_2_S.

In the title compound, (I), the cyclohexene ring adopts a slightly distorted half-chair conformation (puckering parameters Q, θ, and φ = 0.459 (3)Å, 48.9 (2)° and 138.1 (4)°, respectively; Cremer & Pople, 1975) (Fig. 1). The dihedral angles between the mean planes of the thiophene and phenyl rings and the 2-hydroxyphenyl ring are 70.4 (5)° and 12.1 (9)°, respectively. The phenyl and the 2-hydroxyphenyl rings are twisted with respect to each other by 81.0 (6)°. Bond lengths are in normal ranges (Allen *et al.*, 1987). A short intramolecular O2—H2···N1 hydrogen bond is observed. In the crystal, a single weak intermolecular C18–H18···O1 interaction links the molecules into zig-zag chains along [101] which influences the crystal packing (Fig. 2).

### 2. Experimental

To a solution of (2-Amino-4,5,6,7-tetrahydro-benzo[b]thiophen-3-yl)- phenyl-methanone (200 mg, 0.79 mmol) in 10 ml of methanol an equimolar amount of 2-hydroxybenzaldehyde (97 mg, 0.79 mmol) was added dropwise with constant stirring. The mixture was refluxed for 3 hours. A yellow precipitate was obtained. The reaction completion was confirmed by thin layer chromatography. The resulting precipitate was filtered and dried at room temperature overnight. The solid was recrystallized from dichloromethane and the crystals were used as such for x-ray diffraction studies.

### 3. Refinement

All of the H atoms were placed in their calculated positions and then refined using the riding model with Atom—H lengths of 0.93Å (CH); 0.99Å (CH_2_) or 0.82Å (OH). Isotropic displacement parameters for these atoms were set to 1.2 (CH, CH_2_) or 1.5 (OH) times *U*_eq_ of the parent atom. The idealised tetrahedral OH was refined as a rotating group.

### Crystal data

**Table:**

C_22_H_19_NO_2_S	*F*(000) = 760
*M**_r_* = 361.44	*D*_x_ = 1.338 Mg m^−^^3^
Monoclinic, *P*2_1_/*n*	Cu *K*α radiation, λ = 1.54184 Å
*a* = 9.26395 (15) Å	Cell parameters from 5377 reflections
*b* = 14.2886 (2) Å	θ = 4.5–71.3°
*c* = 13.6476 (2) Å	µ = 1.73 mm^−^^1^
β = 96.7581 (15)°	*T* = 173 K
*V* = 1793.97 (5) Å^3^	Block, yellow
*Z* = 4	0.24 × 0.14 × 0.08 mm

### Data collection

**Table:**

Agilent Eos Gemini diffractometer	3462 independent reflections
Radiation source: Enhance (Cu) X-ray Source	3113 reflections with *I* > 2σ(*I*)
Detector resolution: 16.0416 pixels mm^-1^	*R*_int_ = 0.053
ω scans	θ_max_ = 71.5°, θ_min_ = 4.5°
Absorption correction: multi-scan (*CrysAlis PRO* and *CrysAlis RED*; Agilent, 2012)	*h* = −11→10
*T*_min_ = 0.658, *T*_max_ = 1.000	*k* = −17→17
11611 measured reflections	*l* = −16→14

### Refinement

**Table:**

Refinement on *F*^2^	Hydrogen site location: inferred from neighbouring sites
Least-squares matrix: full	H-atom parameters constrained
*R*[*F*^2^ > 2σ(*F*^2^)] = 0.049	*w* = 1/[σ^2^(*F*_o_^2^) + (0.0863*P*)^2^ + 0.6814*P*] where *P* = (*F*_o_^2^ + 2*F*_c_^2^)/3
*wR*(*F*^2^) = 0.142	(Δ/σ)_max_ < 0.001
*S* = 1.06	Δρ_max_ = 1.03 e Å^−^^3^
3462 reflections	Δρ_min_ = −0.26 e Å^−^^3^
237 parameters	Extinction correction: *SHELXL2012* (Sheldrick, 2008), Fc^*^=kFc[1+0.001xFc^2^λ^3^/sin(2θ)]^-1/4^
0 restraints	Extinction coefficient: 0.0024 (4)
Primary atom site location: structure-invariant direct methods	

### Special details

**Table:**

Geometry. All esds (except the esd in the dihedral angle between two l.s. planes) are estimated using the full covariance matrix. The cell esds are taken into account individually in the estimation of esds in distances, angles and torsion angles; correlations between esds in cell parameters are only used when they are defined by crystal symmetry. An approximate (isotropic) treatment of cell esds is used for estimating esds involving l.s. planes.

### Fractional atomic coordinates and isotropic or equivalent isotropic displacement parameters (Å^2^)

**Table:**

	*x*	*y*	*z*	*U*_iso_*/*U*_eq_	
S1	0.55854 (5)	0.11521 (3)	0.69557 (3)	0.02933 (18)	
O1	0.74691 (15)	0.17574 (10)	0.38642 (9)	0.0336 (3)	
O2	0.69355 (16)	0.45879 (10)	0.56596 (11)	0.0364 (4)	
H2	0.6928	0.4014	0.5663	0.055*	
N1	0.61684 (16)	0.29064 (11)	0.62125 (11)	0.0259 (3)	
C1	0.77920 (18)	0.19624 (12)	0.47290 (13)	0.0243 (4)	
C2	0.70603 (18)	0.14805 (12)	0.55066 (13)	0.0248 (4)	
C3	0.70100 (18)	0.04840 (12)	0.56039 (13)	0.0255 (4)	
C4	0.7719 (2)	−0.02126 (13)	0.49863 (15)	0.0312 (4)	
H4A	0.8641	0.0034	0.4832	0.037*	
H4B	0.7103	−0.0316	0.4370	0.037*	
C5	0.7967 (3)	−0.11336 (16)	0.5535 (2)	0.0470 (6)	
H5A	0.8254	−0.1606	0.5086	0.056*	
H5B	0.8757	−0.1059	0.6063	0.056*	
C6	0.6632 (3)	−0.14608 (16)	0.5964 (2)	0.0502 (6)	
H6A	0.6840	−0.2058	0.6288	0.060*	
H6B	0.5858	−0.1560	0.5430	0.060*	
C7	0.6102 (2)	−0.07799 (14)	0.67023 (15)	0.0340 (4)	
H7A	0.5088	−0.0902	0.6767	0.041*	
H7B	0.6658	−0.0868	0.7344	0.041*	
C8	0.62751 (19)	0.02115 (12)	0.63622 (14)	0.0271 (4)	
C9	0.63331 (18)	0.19440 (12)	0.61848 (13)	0.0249 (4)	
C10	0.89550 (18)	0.26513 (12)	0.50409 (13)	0.0243 (4)	
C11	0.9396 (2)	0.32721 (14)	0.43438 (13)	0.0304 (4)	
H11	0.8930	0.3271	0.3702	0.036*	
C12	1.0525 (2)	0.38872 (15)	0.46073 (15)	0.0364 (5)	
H12	1.0806	0.4307	0.4145	0.044*	
C13	1.1242 (2)	0.38818 (15)	0.55585 (16)	0.0369 (5)	
H13	1.2010	0.4292	0.5730	0.044*	
C14	1.0813 (2)	0.32637 (16)	0.62539 (14)	0.0345 (4)	
H14	1.1297	0.3257	0.6891	0.041*	
C15	0.96629 (19)	0.26566 (14)	0.59984 (13)	0.0291 (4)	
H15	0.9364	0.2252	0.6468	0.035*	
C16	0.52973 (19)	0.33090 (13)	0.67519 (14)	0.0272 (4)	
H16	0.4764	0.2942	0.7143	0.033*	
C17	0.51257 (19)	0.43151 (13)	0.67668 (13)	0.0261 (4)	
C18	0.4120 (2)	0.47131 (14)	0.73438 (14)	0.0309 (4)	
H18	0.3603	0.4329	0.7728	0.037*	
C19	0.3894 (2)	0.56679 (15)	0.73455 (15)	0.0359 (5)	
H19	0.3225	0.5924	0.7727	0.043*	
C20	0.4668 (2)	0.62451 (14)	0.67759 (16)	0.0361 (5)	
H20	0.4501	0.6887	0.6769	0.043*	
C21	0.5681 (2)	0.58765 (14)	0.62214 (16)	0.0355 (4)	
H21	0.6205	0.6271	0.5852	0.043*	
C22	0.5925 (2)	0.49131 (13)	0.62120 (14)	0.0281 (4)	

### Atomic displacement parameters (Å^2^)

**Table:**

	*U*^11^	*U*^22^	*U*^33^	*U*^12^	*U*^13^	*U*^23^
S1	0.0347 (3)	0.0245 (3)	0.0310 (3)	−0.00448 (16)	0.01296 (19)	−0.00091 (16)
O1	0.0390 (7)	0.0356 (8)	0.0264 (7)	−0.0060 (6)	0.0048 (5)	−0.0032 (5)
O2	0.0406 (8)	0.0252 (7)	0.0470 (8)	−0.0044 (6)	0.0205 (6)	−0.0017 (6)
N1	0.0265 (7)	0.0222 (7)	0.0295 (8)	−0.0025 (6)	0.0055 (6)	−0.0010 (6)
C1	0.0241 (8)	0.0230 (8)	0.0260 (8)	0.0024 (6)	0.0046 (6)	0.0006 (7)
C2	0.0220 (8)	0.0239 (9)	0.0284 (9)	−0.0022 (7)	0.0027 (6)	−0.0012 (7)
C3	0.0225 (8)	0.0228 (8)	0.0309 (9)	−0.0020 (6)	0.0017 (7)	−0.0018 (7)
C4	0.0310 (9)	0.0273 (9)	0.0359 (10)	0.0022 (7)	0.0063 (7)	−0.0044 (8)
C5	0.0515 (13)	0.0336 (12)	0.0557 (14)	0.0093 (9)	0.0060 (11)	−0.0040 (9)
C6	0.0622 (15)	0.0241 (10)	0.0659 (16)	−0.0026 (10)	0.0144 (12)	−0.0007 (10)
C7	0.0365 (10)	0.0248 (9)	0.0407 (11)	−0.0068 (8)	0.0042 (8)	0.0043 (8)
C8	0.0277 (9)	0.0215 (9)	0.0322 (9)	−0.0019 (7)	0.0034 (7)	−0.0023 (7)
C9	0.0251 (8)	0.0234 (9)	0.0266 (8)	−0.0039 (7)	0.0047 (6)	0.0002 (7)
C10	0.0230 (8)	0.0255 (9)	0.0255 (8)	0.0007 (7)	0.0067 (6)	−0.0001 (7)
C11	0.0321 (9)	0.0353 (10)	0.0238 (8)	−0.0046 (8)	0.0041 (7)	0.0038 (7)
C12	0.0396 (11)	0.0389 (11)	0.0319 (10)	−0.0114 (8)	0.0097 (8)	0.0040 (8)
C13	0.0311 (10)	0.0429 (12)	0.0372 (11)	−0.0131 (8)	0.0064 (8)	−0.0058 (8)
C14	0.0297 (9)	0.0467 (12)	0.0266 (9)	−0.0043 (8)	0.0016 (7)	−0.0025 (8)
C15	0.0281 (9)	0.0347 (10)	0.0253 (9)	−0.0013 (7)	0.0060 (7)	0.0028 (7)
C16	0.0271 (8)	0.0250 (9)	0.0303 (9)	−0.0034 (7)	0.0063 (7)	−0.0006 (7)
C17	0.0246 (8)	0.0245 (9)	0.0288 (9)	−0.0011 (7)	0.0008 (7)	−0.0033 (7)
C18	0.0301 (9)	0.0309 (10)	0.0320 (9)	−0.0011 (7)	0.0051 (7)	−0.0033 (7)
C19	0.0351 (10)	0.0345 (10)	0.0381 (10)	0.0081 (8)	0.0043 (8)	−0.0079 (8)
C20	0.0435 (11)	0.0239 (9)	0.0393 (11)	0.0051 (8)	−0.0021 (8)	−0.0044 (8)
C21	0.0428 (11)	0.0259 (10)	0.0378 (10)	−0.0047 (8)	0.0047 (8)	0.0006 (8)
C22	0.0287 (9)	0.0255 (9)	0.0300 (9)	−0.0029 (7)	0.0027 (7)	−0.0036 (7)

### Geometric parameters (Å, º)

**Table:**

S1—C8	1.7299 (18)	C7—C8	1.505 (3)
S1—C9	1.7432 (18)	C10—C11	1.397 (3)
O1—C1	1.219 (2)	C10—C15	1.392 (3)
O2—H2	0.8200	C11—H11	0.9300
O2—C22	1.351 (2)	C11—C12	1.381 (3)
N1—C9	1.385 (2)	C12—H12	0.9300
N1—C16	1.290 (2)	C12—C13	1.387 (3)
C1—C2	1.493 (2)	C13—H13	0.9300
C1—C10	1.484 (2)	C13—C14	1.388 (3)
C2—C3	1.431 (2)	C14—H14	0.9300
C2—C9	1.377 (2)	C14—C15	1.386 (3)
C3—C4	1.504 (2)	C15—H15	0.9300
C3—C8	1.361 (3)	C16—H16	0.9300
C4—H4A	0.9700	C16—C17	1.447 (3)
C4—H4B	0.9700	C17—C18	1.409 (3)
C4—C5	1.518 (3)	C17—C22	1.409 (3)
C5—H5A	0.9700	C18—H18	0.9300
C5—H5B	0.9700	C18—C19	1.380 (3)
C5—C6	1.504 (4)	C19—H19	0.9300
C6—H6A	0.9700	C19—C20	1.390 (3)
C6—H6B	0.9700	C20—H20	0.9300
C6—C7	1.523 (3)	C20—C21	1.378 (3)
C7—H7A	0.9700	C21—H21	0.9300
C7—H7B	0.9700	C21—C22	1.395 (3)
			
C8—S1—C9	91.53 (9)	C2—C9—N1	124.08 (16)
C22—O2—H2	109.5	C11—C10—C1	119.12 (16)
C16—N1—C9	122.47 (15)	C15—C10—C1	121.26 (16)
O1—C1—C2	119.82 (16)	C15—C10—C11	119.53 (17)
O1—C1—C10	121.62 (16)	C10—C11—H11	120.0
C10—C1—C2	118.49 (15)	C12—C11—C10	120.01 (18)
C3—C2—C1	123.18 (15)	C12—C11—H11	120.0
C9—C2—C1	123.70 (16)	C11—C12—H12	119.8
C9—C2—C3	113.09 (16)	C11—C12—C13	120.33 (18)
C2—C3—C4	125.81 (16)	C13—C12—H12	119.8
C8—C3—C2	112.33 (16)	C12—C13—H13	120.0
C8—C3—C4	121.83 (17)	C12—C13—C14	119.94 (18)
C3—C4—H4A	109.6	C14—C13—H13	120.0
C3—C4—H4B	109.6	C13—C14—H14	120.0
C3—C4—C5	110.36 (17)	C15—C14—C13	119.98 (18)
H4A—C4—H4B	108.1	C15—C14—H14	120.0
C5—C4—H4A	109.6	C10—C15—H15	119.9
C5—C4—H4B	109.6	C14—C15—C10	120.19 (17)
C4—C5—H5A	109.2	C14—C15—H15	119.9
C4—C5—H5B	109.2	N1—C16—H16	119.1
H5A—C5—H5B	107.9	N1—C16—C17	121.89 (16)
C6—C5—C4	112.2 (2)	C17—C16—H16	119.1
C6—C5—H5A	109.2	C18—C17—C16	119.34 (17)
C6—C5—H5B	109.2	C22—C17—C16	121.99 (16)
C5—C6—H6A	108.8	C22—C17—C18	118.67 (17)
C5—C6—H6B	108.8	C17—C18—H18	119.7
C5—C6—C7	113.75 (19)	C19—C18—C17	120.70 (18)
H6A—C6—H6B	107.7	C19—C18—H18	119.7
C7—C6—H6A	108.8	C18—C19—H19	120.1
C7—C6—H6B	108.8	C18—C19—C20	119.81 (18)
C6—C7—H7A	109.6	C20—C19—H19	120.1
C6—C7—H7B	109.6	C19—C20—H20	119.7
H7A—C7—H7B	108.2	C21—C20—C19	120.70 (18)
C8—C7—C6	110.05 (17)	C21—C20—H20	119.7
C8—C7—H7A	109.6	C20—C21—H21	119.9
C8—C7—H7B	109.6	C20—C21—C22	120.24 (19)
C3—C8—S1	112.29 (14)	C22—C21—H21	119.9
C3—C8—C7	125.78 (17)	O2—C22—C17	122.26 (17)
C7—C8—S1	121.89 (14)	O2—C22—C21	117.89 (17)
N1—C9—S1	125.12 (13)	C21—C22—C17	119.85 (18)
C2—C9—S1	110.74 (13)		
			
O1—C1—C2—C3	53.9 (2)	C8—C3—C4—C5	−20.5 (3)
O1—C1—C2—C9	−124.32 (19)	C9—S1—C8—C3	−1.22 (14)
O1—C1—C10—C11	18.8 (3)	C9—S1—C8—C7	176.49 (16)
O1—C1—C10—C15	−157.73 (17)	C9—N1—C16—C17	−179.41 (16)
N1—C16—C17—C18	177.77 (17)	C9—C2—C3—C4	−178.91 (16)
N1—C16—C17—C22	−1.6 (3)	C9—C2—C3—C8	−1.2 (2)
C1—C2—C3—C4	2.7 (3)	C10—C1—C2—C3	−123.18 (18)
C1—C2—C3—C8	−179.58 (16)	C10—C1—C2—C9	58.6 (2)
C1—C2—C9—S1	178.63 (13)	C10—C11—C12—C13	1.1 (3)
C1—C2—C9—N1	1.4 (3)	C11—C10—C15—C14	−1.0 (3)
C1—C10—C11—C12	−176.81 (18)	C11—C12—C13—C14	−0.8 (3)
C1—C10—C15—C14	175.54 (17)	C12—C13—C14—C15	−0.4 (3)
C2—C1—C10—C11	−164.18 (16)	C13—C14—C15—C10	1.3 (3)
C2—C1—C10—C15	19.2 (2)	C15—C10—C11—C12	−0.2 (3)
C2—C3—C4—C5	156.98 (18)	C16—N1—C9—S1	−7.9 (3)
C2—C3—C8—S1	1.60 (19)	C16—N1—C9—C2	168.92 (17)
C2—C3—C8—C7	−176.01 (17)	C16—C17—C18—C19	−177.65 (17)
C3—C2—C9—S1	0.30 (19)	C16—C17—C22—O2	−2.3 (3)
C3—C2—C9—N1	−176.90 (16)	C16—C17—C22—C21	177.57 (17)
C3—C4—C5—C6	48.4 (3)	C17—C18—C19—C20	−0.3 (3)
C4—C3—C8—S1	179.39 (13)	C18—C17—C22—O2	178.33 (17)
C4—C3—C8—C7	1.8 (3)	C18—C17—C22—C21	−1.8 (3)
C4—C5—C6—C7	−60.2 (3)	C18—C19—C20—C21	−1.2 (3)
C5—C6—C7—C8	38.5 (3)	C19—C20—C21—C22	1.1 (3)
C6—C7—C8—S1	172.38 (16)	C20—C21—C22—O2	−179.69 (18)
C6—C7—C8—C3	−10.2 (3)	C20—C21—C22—C17	0.4 (3)
C8—S1—C9—N1	177.66 (16)	C22—C17—C18—C19	1.7 (3)
C8—S1—C9—C2	0.50 (14)		

### Hydrogen-bond geometry (Å, º)

**Table:**

*D*—H···*A*	*D*—H	H···*A*	*D*···*A*	*D*—H···*A*
O2—H2···N1	0.82	1.92	2.641 (2)	146
C18—H18···O1^i^	0.93	2.51	3.436 (2)	174

Symmetry code: (i) *x*−1/2, −*y*+1/2, *z*+1/2.
